# The discrepancy in triggered electromyography responses between fatty filum and normal filum terminale

**DOI:** 10.1186/s12893-024-02351-0

**Published:** 2024-02-16

**Authors:** Yizi Cai, Rui Wang, Junlu Wang, Qijia Zhan, Min Wei, Bo Xiao, Qiang Wang, Wenbin Jiang

**Affiliations:** 1 Department of Neonatology, Shanghai Children’s Hospital, School of Medicine, Shanghai Jiao Tong University, Shanghai, China; 2 Department of Neurosurgery, Shanghai Children’s Hospital, School of Medicine, Shanghai Jiao Tong University, 355 Luding Road, Shanghai, 200062 China; 3 Department of Neurosurgery, Shanghai Children’s Medical Center, School of Medicine, Shanghai Jiao Tong University, Shanghai, China; 4 College of Computer and Network Engineering, Shanxi Datong University, Datong, China

**Keywords:** Filum terminale, Electrophysiology, Electromyography, Intraoperative neurophysiological monitoring

## Abstract

**Background:**

Functional role of filum terminale (FT) was not well studied though it contains structure basis for nerve impulse conduction. We aimed to explore the possible functions of the FT from the perspective of triggered electromyography (EMG) during surgery.

**Methods:**

We retrospectively reviewed intraoperative neurophysiological monitoring data from pediatric patients who underwent intradural surgeries at the lumbar level in Shanghai Children’s. Hospital from January 2018 to March 2023. Altogether 168 cases with complete intraoperative neurophysiological recordings of the FT were selected for further analysis. Triggered EMG recordings of the filum originated from two main types of surgeries: selective dorsal rhizotomy (SDR) and fatty filum transection.

**Results:**

96 cases underwent SDR and 72 cases underwent fatty filum transection. Electrical stimulation of the FT with fatty infiltration did not elicit electromyographic activity in the monitored muscles with the maximum stimulus intensity of 4.0 mA, while the average threshold for FT with normal appearance was 0.68 mA, and 89 out of 91 FT could elicit electromyographic responses in monitored channels. The threshold ratio of filum to motor nerve roots at the same surgical segment was significantly higher in patients with fatty filum, and a cut-off point of 21.03 yielded an area under curve of 0.943, with 100% sensitivity and 85.71% specificity.

**Conclusion:**

Filum with normal appearance can elicit electromyographic activity in the lower limbs/anal sphincter similar to the performance of the cauda equina nerve roots. The threshold of fatty filum is different from that of normal appearing FT. Triggered EMG plays an important role in untethering surgeries.

**Supplementary Information:**

The online version contains supplementary material available at 10.1186/s12893-024-02351-0.

## Introduction

Filum terminale (FT) is an elastic fibrous structure that connects the conus medullaris to the coccyx [1–5]. Normal internal FT can be easily distinguished from the cauda equina nerve roots by its pearly white color under a surgical microscope. However, in some circumstances, the appearance of filum would be changed with fat infiltration if some failure happened in late phase of secondary neurulation and were defined as the lipoma of FT. The lipoma of FT accounts for a proportion of tethered cord syndrome, and surgical resection is recommended if it is classified as Grade 1–3 according to the classification suggested by Blount et al., which in detail, either present with abnormal position of conus medullaris or the clinical urinary/fecal disorders [6].

It is generally considered that FT is a remnant of the spinal cord with no other function than an anchor stabilizing the caudal cord [7, 8]. However, studies of the normal FT have revealed that it contains neuronal structures in addition to collagen under transmission electron microscopy, hence, the FT has the structural basis for neuronal function [3, 9–11]. Recent research has shown the presence of Remak cells and Ruffini corpuscles in the FT, suggesting that it might be part of the complex somatosensory-motor system of the spine, and could contribute to non-physiological pulling forces caused by excessive spinal activity [11]. The sensory tissue in the FT may play a crucial role in the back pain symptom that occurs in many spinal diseases. Based on the background, we believe that the FT might serve some neuronal functions. As a structure formed following spinal cord degeneration, it is possible that electrical stimulation of the FT may activate the neural network within the spinal cord. Previous studies have reported that direct electrical stimulation of the FT can induce electromyographic activity in anal sphincter, exhibiting responses similar to those of the sacral nerve roots [11, 12]. What’s more, the intact nerve cell bodies, axons and synapses within the FT in adult cats and monkeys suggested the anatomical basis for nerve impulse conduction and transmission [13].

It is thus reasonable to speculate that the pathological change of FT would not only affect the appearance, but might also alter the function of FT. Nonetheless, whether the functional role of FT in fatty filum diminished remained unknown. To date, there has been limited researches on the functional role of the FT in human beings [14]. Therefore, we reviewed the intraoperative neurophysiological recordings of the responses after the electrical stimulation of FT in our center, hoping to (1) explore the possible functions of the FT from the perspective of triggered electromyography (EMG), (2) find out whether the neuronal function of FT would change after the pathological change and (3) verify the safety of filum transection surgery.

## Methods and materials

### Data collection

This study received ethical approval from the Ethics Committee of Shanghai Children’s Hospital with approval numbers 2020R069-E02 and 2022R130-E01. The intraoperative neurophysiological monitoring data were collected from pediatric patients who underwent intradural surgeries at the lumbar level (below the conus medullaris) in Shanghai Children’s Hospital from January 2018 to March 2023. We screened for cases with complete intraoperative neurophysiological recordings and selected those with triggered EMG of the FT for further analysis, excluding patients with complex spinal dysraphism such as spinal meningocele, spinal cord lipoma, or spinal cord meningocele. Triggered EMG recordings of the filum originated from two main types of surgeries: selective dorsal rhizotomy (SDR) and fatty filum transection. All FT were classified into two categories based on their intraoperative appearance: normal or lipoma of FT. Patients with a normal filum appearance were children with spastic cerebral palsy who underwent SDR, while patients with abnormal filum appearance were mostly children who underwent filum transection due to fatty filum who met the surgical indication depicted by Blount et al [6], but also some cases with spastic cerebral palsy.

### Brief description of SDR

SDR is performed in children suffering from spastic cerebral palsy who met the surgical indications [15]. During the surgery, the child was placed in a prone position with the head lowered, and the surgical incision was typically made at the L2-L3 interspace. After laminectomy, we made an incision of about 1.2 cm in the midline of the dura mater. With guidance from neurophysiological monitoring, we carefully separated the cauda equina and FT in the surgical area, and tested the filum and nerve roots with single-pulse electrical stimulation one by one, with all neurophysiological data recorded [16–19].

### Surgical technique for filum transection

For patients diagnosed with fatty filum confirmed by magnetic resonance imaging with either a descend conus or urinary/bowel symptoms, we would recommend the filum transection. Before the operation, the patient was placed in a prone position with the head lowered. The surgical incision was generally made at the lower lumbar level to avoid the damage to conus medullaris [20]. After laminectomy and midline durotomy, we differentiate and separate the FT structure under a microscope. Before performing electrical stimulation to the FT, we confirmed the configuration of the neurophysiological monitoring system (usually by stimulating the ventral motor roots at the level of incision). After the confirmation, the FT was tested by single-pulse electrical stimulation. The filum would then be transected under the guidance of neurophysiological monitoring.

### Setting of intraoperative neurophysiological monitoring

The neurophysiological monitoring system used in SDR and untethering surgeries was CADWELL-CASCADE. This study focuses on triggered EMG, so the related settings are mainly described. During SDR, 15 muscle groups were monitored by needle electrodes, including bilateral adductors, hamstrings, quadriceps, tibialis anterior, medial and lateral gastrocnemius, peroneus longus and anal sphincter. While in the FT section surgery, since additional SSEP and MEP monitoring were required, the number of channels for trigger EMG monitoring reduced to 9 channels, including bilateral quadriceps, hamstrings, tibialis anterior, gastrocnemius, and anal sphincter.

When performing electrical stimulation on the FT/nerve root that needed to be tested, we used a bipolar stimulator. The parameters for electrical stimulation were single-pulse electrical stimulation with a pulse width of 0.2 msec, with an initial stimulation intensity of 0 mA and an intensity increase step width of 0.01 mA. We set an endpoint for the stimulation of the fiber we test, which will stop the electrical stimulation in any of the following situations: (1) stable compound muscle action potential (CMAP) ≥ 20µV appeared in the anal sphincter monitoring channel during the stimulation process; (2) stable CMAP ≥ 200µV appeared on any monitoring channel other than anal sphincter during the stimulation process; (3) the stimulation current reached 4.0 mA, but neither situation 1 nor 2 occurred. In order to avoid over-stimulation and damage to the FT and detected nerve roots, we would stop the stimulation at 4.0 mA. The stimulation intensity at endpoint was defined as the threshold.

### Anesthetic setting during intraoperative monitoring

All surgeries were done under general anesthesia. To ensure the stability of monitoring, the temperature of patients were maintained between 36.0 and 37.0 ℃, minimum alveolar concentration of sevoflurane inhalation was kept at 0.5 during the surgery, and muscle relaxants were prohibited after the intubation [21].

### Standardization of intraoperative monitoring data and the threshold ratio of filum to motor nerve root

To compare the triggered EMG data of FT among different patients, as well as to visualize the spreading of channels and the channel with highest EMG amplitude, we performed data standardization to the neurophysiological data using Min-Max normalization. Specifically, we identified the minimum and maximum values of triggered EMG amplitudes in the monitoring channels after the stimulation, and then converted each original amplitude value x to a value x’ in the range of [0,1] through min-max normalization. The formula was: X’ = (X-Xmin) / (Xmax-Xmin). After standardization, the results were expressed in a heat map and then sorted to exhibit the channel with highest EMG amplitude.

As the surgical approach in SDR was uniformly L2-L3 interlaminar, but the surgical approach in untethering surgeries varied. To solve the difference, we calculated the threshold ratio of filum to motor nerve roots at the same segment. The formula was Threshold Ratio = Threshold _filum_/ Threshold _motor nerve root_. It has to be mentioned that the threshold of motor nerve root equals the average of thresholds on both sides at surgical area.

### Statistical analysis

All statistical analyses were conducted using SPSS (version 25.0, IBM Corp.). The Shapiro-Wilk test was used to evaluate the normality of continuous data with *p* value set at 0.05. Continuous data with normal distribution were presented as mean ± SD and variables deviated from a normal distribution pattern were presented as median (Q1, Q3). Continuous variables were compared between groups using Mann-Whitney or t-tests, as appropriate. Categorical variables were analyzed using chi-square test, and Fisher exact test was applied whenever appropriate. Receiver operating characteristic (ROC) curve was generated, and the area under curve, sensitivity and specificity were calculated. A *p*-value less than 0.05 was considered statistically significant.

## Results

**Fig. 1:**
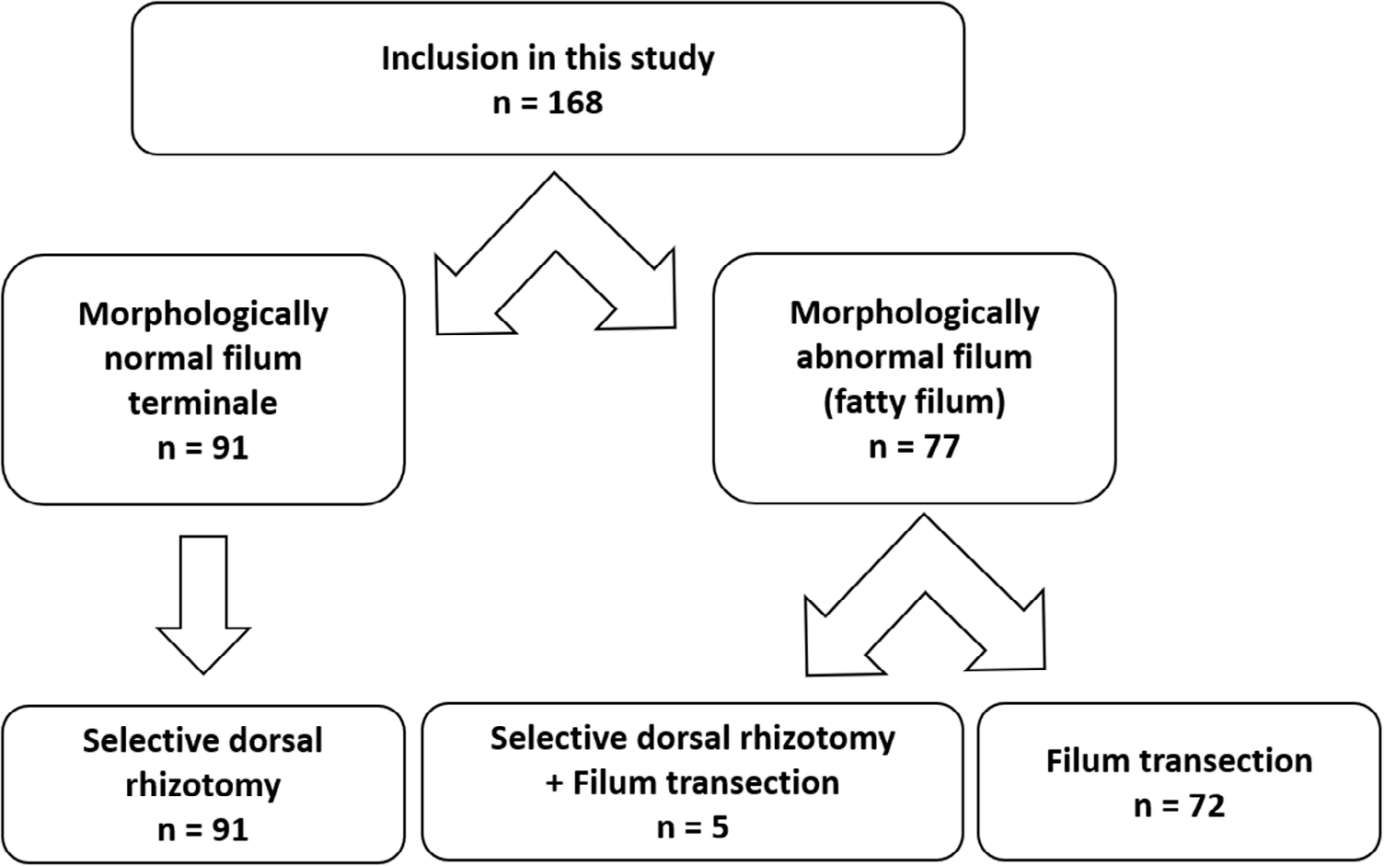
Flowchart of patients included in this study

We reviewed a total of 168 cases with complete triggered EMG recordings of FT, including 96 cases recorded during SDR, and 72 cases recorded during filum transection (Fig. 1). In 5 children suffering from spastic cerebral palsy, they were also found to have fatty filum on pre-operative lumbar and sacral magnetic resonance imaging, and these 5 patients underwent both SDR surgery and filum transection under electrophysiological monitoring.

**Fig. 2:**
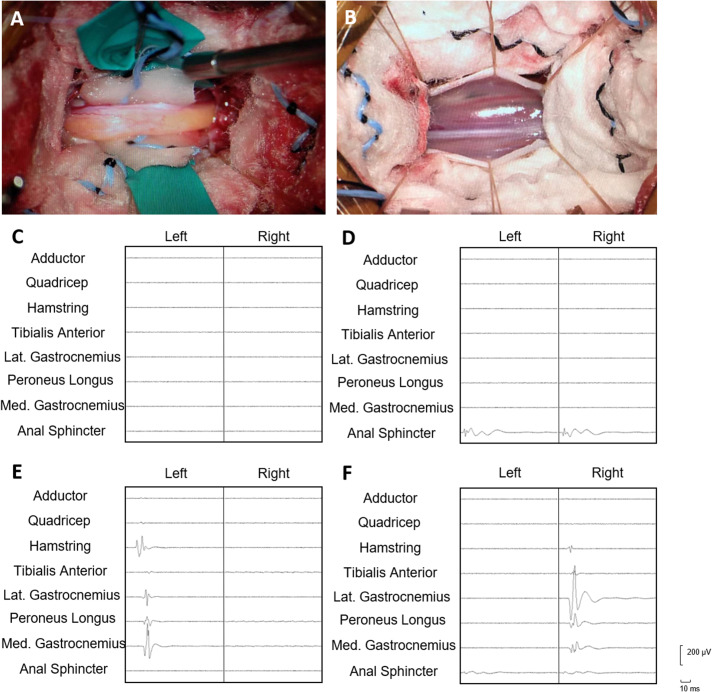
Morphology of fatty filum and normal filum terminale, and the triggered electromyography (EMG) of filum. (**A**) Morphology of fatty filum under microscopy. (**B**) Morphology of normal filum under microscopy. (**C**) Triggered EMG showing no muscle contraction in any monitored channel. (**D**) Triggered EMG showing EMG response only in anal sphincter. (**E**) Triggered EMG showing EMG response in muscles of left lower extremity (hamstring, gastrocnemius and peroneus longus). (**F**) Triggered EMG showing EMG response in muscles of right lower extremity (hamstring, gastrocnemius and peroneus longus) and anal sphincter

**Fig. 3:**
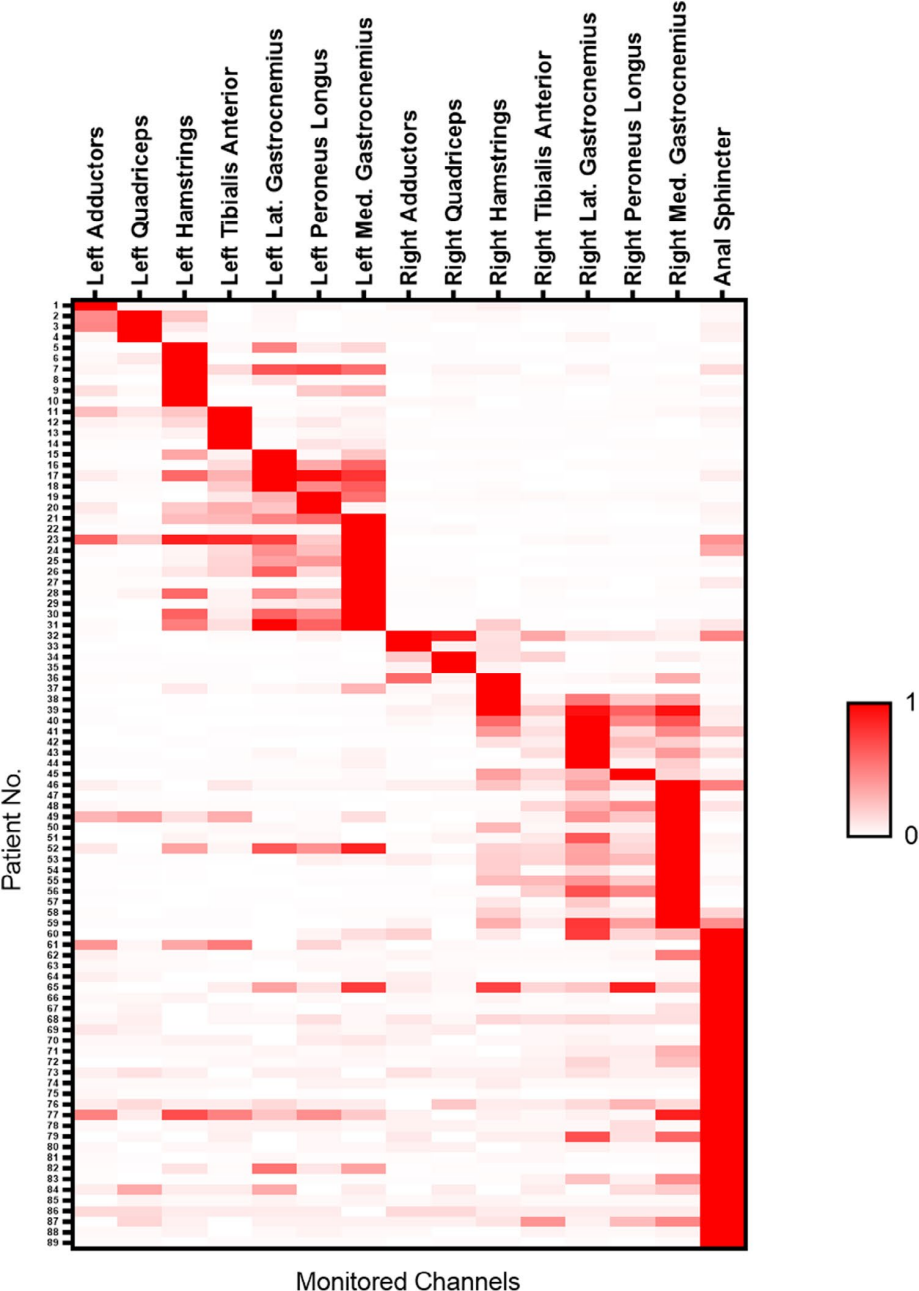
Heatmap of triggered EMG in filum terminale with electromyographical responses (after sorting)

77 abnormal FT were measured with an average diameter of 1.7 mm during surgeries (Fig. 2-A), these 77 FT with fat infiltration were obtained from 43 males and 34 females with a median age of 60.0 months (Table 1). 91 normal FT were measured during surgery with a diameter less than 1 mm (Fig. 2-B). These 91 normal FT were obtained from 69 males and 22 females with a median age of 69.8 months. Electrical stimulation of the FT with fatty infiltration did not elicit electromyographic activity in the monitored muscles with a maximum stimulating current of 4.0 mA (Fig. 2-C), while the median threshold of FT with normal appearance was 0.68 mA (0.33, 1.79), and 89 out of 91 FT could elicit electromyographic responses in the monitored channel. The responses were observed in 23.9% only in the anal sphincter, 46.7% only in leg muscles, and 28.3% in both anal sphincter and leg muscles (Figs. 2D-F and 3). After both SDR and untethering procedures, no obvious urinary/bowel complications happened.

**Table 1:** Demographic details in this study

Variables	Morphologically normal filum	Morphologically abnormal filum	
Filum derivation	SDR (*n* = 91)	SDR (*n* = 5)	Filum transection (*n* = 72)
Age (Mon, median)	69.8 (59.8, 82.6)	69.3 (57.1, 104.1)	54.6 (17.1, 96.0)
Gender (female) (%)	22 (24.2%)	3 (60.0%)	31 (43.1%)
Surgical approach (%)			
L2/3	91 (100%)	5 (100%)	12 (16.7%)
L3/4	0 (0%)	0 (0%)	25 (34.7%)
L4/5	0 (0%)	0 (0%)	35 (48.6%)
Threshold of motor nerve roots at surgical level (mA, median)	0.11 (0.07, 0.14)	0.10 (0.07, 0.11)	0.13 (0.08, 0.16)
Threshold of filum (mA, median)	0.68 (0.33, 1.80)	4.0	4.0^****^
The threshold ratio of filum to motor nerve roots at the same surgical level	5.4 (2.9, 15.6)	36.4 (25.6, 51.5)	32.1^****^ (25.0, 50.0)
Reaction to electrical stimulation (%)			
Only in anal sphincter	22 (23.9%)	0 (0%)	0 (0%)
Only in leg muscles	43 (46.7%)	0 (0%)	0 (0%)
Both anal sphincter and leg muscles	26 (28.3%)	0 (0%)	0 (0%)

^****^: significantly different when compared with morphologically normal filum (*p* < 0.0001)

To further explore the threshold of the FT, we compared the threshold of the motor nerve roots at the surgical entry with the threshold of the FT. While the surgical approach in SDR was uniformly L2-L3 interlaminar, the surgical approach in untethering surgeries varied (Table 1). To eliminate this factor, we compared the threshold of filum with the threshold of motor nerve roots in the same segment. The threshold ratio of filum to motor nerve roots was 5.4 in median in patients with normal filum, while in five patients with both cerebral palsy and fatty filum, the median ratio was 36.4. In patients with fatty filum only, the ratio was 32.1 times in median.

**Fig. 4:**
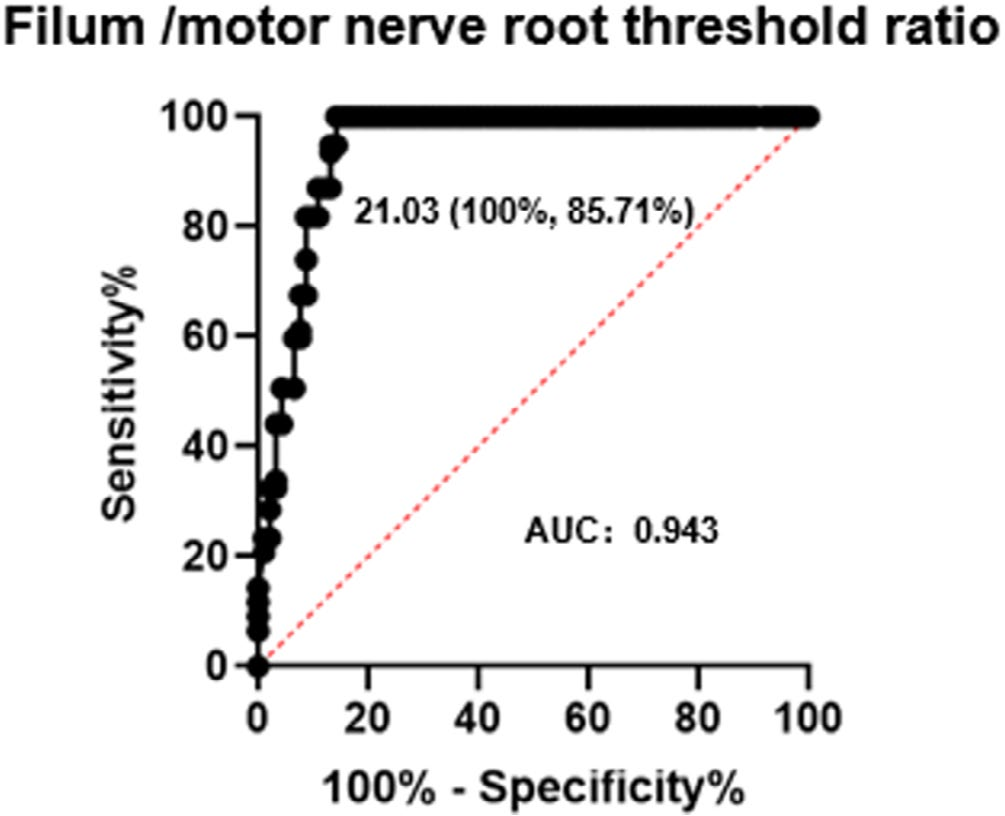
Receiver Operating Characteristic curve comparing neurophysiological differences (threshold ratio of the filum to motor nerve roots at the same surgical level) between fatty filum and normal filum. The curve represents the relationship between sensitivity and specificity at various threshold ratios of the filum to motor nerve roots. The Area Under the Curve (AUC) is a quantitative measure of the curve’s discriminative ability. The AUC was 0.943 (*p* < 0.0001), the sensitivity was 100%, and the specificity was 85.71% if the cut-off point was taken as 21.03

We presented the ROC curve, a graphical representation illustrating the neurophysiological difference between fatty filum and normal filum in Fig. 4. This curve evaluated the threshold ratio of the filum to motor nerve roots at the same surgical level and provided insights into the discriminative ability of our neurophysiological measurements. The AUC, a quantitative measure of the ROC curve’s performance, was reported as 0.943. This metric served as an indicator of the diagnostic accuracy of our filum stimulation threshold determination. The dotted line on the ROC curve denoted the cut-off point for significance, set at 21.03. At this point, the sensitivity was 100%, and the specificity was 85.71%. The ROC analysis demonstrated the effectiveness of our approach in distinguishing between fatty filum and normal filum based on the threshold ratio of the filum to motor nerve roots.

## Discussion

In this study, FT with normal appearances were derived from children with spastic cerebral palsy, while FT with abnormal fat infiltration consisted of children both diagnosed with cerebral palsy and fatty filum. We were not able to obtain electrophysiological data of FT in normal children, therefore, we selected children with normal FT appearance who underwent SDR guided by intraoperative neurophysiological monitoring. The accessibility of FT stimulation during SDR made these children the best candidates serving as objects for the exploration of filum. At the same time, we also retrospectively reviewed the intraoperative electrical stimulation records of patients with fatty filum because we believed that in these patients, the normal function of FT might have been altered. They would be ideal comparisons with normal FT.

The findings of the current study indicated that the normal FT responded similarly to the cauda equina nerve roots when stimulated by single-pulse electrical stimulation. Previous studies have suggested two possible reasons for this [22]. The first possible explanation is that there exists a connection between the FT and adjacent cauda equina roots, similar to the Rasmussen connection of the seventh and eighth pairs of cranial nerves. This might be the structural basis for the evoked EMG of lower limb/anal muscles under single-pulse electrical stimulation. The second is that the FT itself has neurons that can activate the spinal sensory input neural network under the effect of electrical stimulation. The FT contains Remak cells and Ruffini corpuscles, as well as many mature nerve markers [11]. These structures allow the FT to respond to electrical stimulation, which in detail, eliciting EMG responses in muscles of lower limbs or anal sphincter.

We did not have access to the biopsy of normal FT, but previous studies have shown that there are positive markers such as S100 and GFAP in normal FT in cadavers [10, 22, 23]. Pathological immunohistochemical examination of fatty filum in our series revealed that, though the fat infiltrated the filum, mature neural markers such as GFAP and S100 could still be found in FT (Supplementary Fig. 2). GFAP is a marker expressed in Schwann cells, radial glia cells, and astrocytes, while S100 is a biomarker expressed in Schwann cells and astrocytes. However, the diseased filaments could not induce electromyographic responses in the lower limbs/anal sphincter muscles after electrical stimulation. We speculate the possible explanation might be: (1) Adipose infiltration increases the resistance of the filum, so a larger electric stimulation intensity might be required to activate the spinal cord neural network. Previous studies have suggested that diseased filum requires an electric current stimulus of up to 6.0 mA to induce contraction of the paravertebral muscles [11], while the maximum stimulus current in this study was 4.0 mA, which may be far below its threshold. Further animal experiments/clinical studies may be needed to clarify this question. (2) The presence of adipose cells disrupts the continuity of the original neural structure, so fatty filum cannot function like normal one. (3) The spinal cord tethering caused by the diseased filum structure has resulted in the disruption of the spinal cord distal network. Regardless of the reasons, the diseased filum has lost original structure and function and require surgical transection.

In the current study, we explored the threshold of normal filum and fatty filum, as well as the threshold ratio of FT to the motor roots at same segment. The results showed that the threshold of fatty filum is much higher than normal one, and the median filum/motor nerve root ratio was 6 times higher than the ratio of normal filum. Nonetheless, the actual threshold of fatty filum might be much higher than 4.0 mA, the upper limit set in our center, as no EMG responses could be found at the stimulus intensity at 4.0 mA. This confirmed the findings in previous study [24]. One thing should be noticed is that the stimulation of filum during SDR and untethering surgeries may not be at the same segment. To solve this, we use the threshold ratio. It has been shown that the difference between the threshold of the motor root and the FT in diseased FT structures can be up to 100 times [25]. Although the difference in the ratio we obtained in this study was smaller than what is reported in previous studies, the reasons for this might be: (1) constant voltage stimulation was used in previous research, while we use constant current to stimulate the filum. The difference between constant voltage/current stimulation may cause deviation in the threshold difference. (2) To avoid damage, we restricted the upper limit of the stimulation current to 4 mA, which may be lower than the threshold that fatty filum should have, thereby narrowing the ratio. However, our results can still serve as a guide in FT transection surgeries.

It has been suggested that there are degenerated axons and atrophied myelin sheaths in the structure of the FT, which may be residual fibers from early neural development. The FT might be the residual tissue that degenerated during the development of the spinal cord, or a degenerated neural structure in the process of biological evolution. Therefore, we speculate that the induced electromyographic response of the FT we have discovered may be a degenerated and related network of spinal cord input. In addition to the aforementioned evoked EMG manifestations, researches have shown that normal FT contain developmental neuro-markers, and it is believed that the filum contain stem cells with neurogenic potential [26, 27]. This may provide a therapeutic foundation for clinical treatment related to neural stem cells. Therefore, we have reasons to believe that the FT may not only be a remnant or residual, but also a structure that we need to further explore.

The fatty filum either causing the bladder/bowel syndrome or tethering the spinal cord had lost their function compared with most of the filum with normal appearance. The EMG responses caused by the electrical stimulation of filum may be arise from the degenerated spinal circuit probably existed in ancient times. Transecting the fatty filum under the supervision of intraoperative monitoring is safe. It is reported in some center that tight filum without fat infiltration can cause bladder/bowel syndrome or back pain, and thus need surgical treatment [28, 29]. After the operation, symptoms would be relieved. It is speculated that electrical responses of tight filum without fat infiltration might react similarly as the fatty filum and could thus be cut safely. However, currently there’s limited studies about this. Further researches could be conducted to figure out and set the foundation for the indication of filum transection in spina bifida occulta.

## Limitations

Several limitations should be acknowledged when interpreting the findings of our study. Firstly, our investigation primarily focuses on the functional aspects of the filum, lacking comprehensive molecular, immunohistochemical, and ultrastructural studies of normal FT structure. The constraints of our medical center’s resources hindered the execution of such detailed examinations. Secondly, the upper limit of stimulus intensity for single-pulse electrical stimulation of the FT was set at 4.0 mA, a decision informed by clinical experience and existing literature. This limitation, coupled with a restricted number of monitored muscle groups, potentially constrained the depth of our exploration into the FT’s functional dynamics.

Additionally, the use of normal FT samples were derived from patients with spastic cerebral palsy, rather than the general population, introduces uncertainties regarding the generalizability of our findings. The diversity in stimulated FT segments across patients during surgeries, exemplified by variations in the L2-L3 and L4-L5 segments, adds an additional layer of complexity. While our study represents the largest recorded investigation of filament-induced electromyography to date, it is crucial to acknowledge that our findings do not conclusively establish the clinically relevant impact of normal FT on lower limb muscle and anal sphincter functioning. Furthermore, our study does not definitively demonstrate the utility of FT stimulation threshold determination in enhancing the safety of FT transection procedures, especially in cases involving non-fatty filum.

These limitations underscore the need for future research endeavors to address these gaps in understanding and refine methodologies for a more nuanced comprehension of FT function and its clinical implications.

## Conclusions

Filum with normal appearance can elicit electromyographic activity in the lower limbs/anal sphincter similar to the performance of the cauda equina nerve roots, while pathological FT cannot elicit electromyographic activity in the lower limbs/anal sphincter under electrical stimulation. The threshold of fatty filum is different from that of normal appearing FT, and the difference between fatty FT and the same segmental motor root is greater than that of normal FT and the same segmental motor root. Triggered EMG plays an important role in filum transection.

### Electronic supplementary material

Below is the link to the electronic supplementary material.

Supplementary Material 1

## Data Availability

The datasets used and analyzed during this study are available from the corresponding author on reasonable request.

## References

[1] McElroy A, Klinge P, Sledge D, Donahue J, Glabman R, Rashmir A (2021). Evaluation of the Filum Terminale in Hereditary equine Regional dermal asthenia. Vet Pathol.

[2] Fontes R, Saad F, Soares M, de Oliveira F, Pinto F, Liberti E (2006). Ultrastructural study of the filum terminale and its elastic fibers. Neurosurgery.

[3] Saker E, Henry B, Tomaszewski K, Loukas M, Iwanaga J, Oskouian R, Tubbs R (2017). The filum terminale internum and externum: a comprehensive review. J Clin Neuroscience: Official J Neurosurgical Soc Australasia.

[4] De Vloo P, Monea A, Sciot R, van Loon J, Van Calenbergh F (2016). The Filum Terminale: a cadaver study of anatomy, histology, and Elastic properties. World Neurosurg.

[5] Klinge P, Srivastava V, McElroy A, Leary O, Ahmed Z, Donahue J, Brinker T, De Vloo P, Gokaslan Z (2022). Diseased Filum Terminale as a cause of tethered cord syndrome in Ehlers-Danlos syndrome: histopathology, Biomechanics, Clinical Presentation, and outcome of Filum Excision. World Neurosurg.

[6] Blount J, Elton S (2001). Spinal lipomas. NeuroSurg Focus.

[7] Jiang Q, Tao B, Gao G, Sun M, Wang H, Li J, Wang Z, Shang A (2022). Filum Terminale: a Comprehensive Review with Anatomical, pathological, and Surgical considerations. World Neurosurg.

[8] Gamble H (1971). Electron microscope observations upon the conus medullaris and filum terminale of human fetuses. J Anat.

[9] Jang H, Cho K, Chang H, Jin Z, Rodriguez-Vazquez J, Murakami G (2016). The Filum Terminale Revisited: a histological study in human fetuses. Pediatr NeuroSurg.

[10] Choi B, Kim R, Suzuki M, Choe W (1992). The ventriculus terminalis and filum terminale of the human spinal cord. Hum Pathol.

[11] Klinge P, McElroy A, Leary O, Donahue J, Mumford A, Brinker T, Gokaslan Z (2022). Not just an Anchor: the human Filum Terminale contains Stretch sensitive and nociceptive nerve endings and responds to Electrical Stimulation with Paraspinal muscle activation. Neurosurgery.

[12] Cabrera J, Vigueras S, Muñoz R, López E (2020). Double neurophysiological certification of the filum terminale during sectioning surgery in pediatric population. Surg Neurol Int.

[13] Miller C (1968). The ultrastructure of the conus medullaris and filum terminale. J Comp Neurol.

[14] Durdağ E, Börcek P, Öcal Ö, Börcek A, Emmez H, Baykaner M (2015). Pathological evaluation of the filum terminale tissue after surgical excision. Child’s Nerv System: ChNS : Official J Int Soc Pediatr Neurosurg.

[15] Nicolini-Panisson R, Tedesco A, Folle M, Donadio M, Selective dorsal rhizotomy in cerebral palsy: selection criteria and postoperative physical therapy protocols (2018). Revista paulista de pediatria: orgao oficial da Sociedade de Pediatria de Sao Paulo.

[16] Jiang W, Jiang S, Yu Y, Zhan Q, Wei M, Mei R, Chen F, Guo Y, Xiao B (2022). Improvement of the gait pattern after selective dorsal rhizotomy derives from changes of kinematic parameters in the sagittal plane. Front Pead.

[17] Xiao B, Constatntini S, Browd S, Zhan Q, Jiang W, Mei R (2020). The role of intra-operative neuroelectrophysiological monitoring in single-level approach selective dorsal rhizotomy. Child’s Nerv System: ChNS : Official J Int Soc Pediatr Neurosurg.

[18] Zhan Q, Tang L, Wang Y, Xiao B, Shen M, Jiang S, Mei R, Lyu Z (2019). Feasibility and effectiveness of a newly modified protocol-guided selective dorsal rhizotomy via single-level approach to treat spastic hemiplegia in pediatric cases with cerebral palsy. Child’s Nerv System: ChNS : Official J Int Soc Pediatr Neurosurg.

[19] Zhan Q, Yu X, Jiang W, Shen M, Jiang S, Mei R, Wang J, Xiao B (2020). Whether the newly modified rhizotomy protocol is applicable to guide single-level approach SDR to treat spastic quadriplegia and diplegia in pediatric patients with cerebral palsy?. Child’s Nerv System: ChNS : Official J Int Soc Pediatr Neurosurg.

[20] Lalgudi Srinivasan H, Valdes-Barrera P, Agur A, Soleman J, Ekstein M, Korn A, Vendrov I, Roth J, Constantini S (2021). Filum terminale lipomas-the role of intraoperative neuromonitoring. Child’s Nerv System: ChNS : Official J Int Soc Pediatr Neurosurg.

[21] Sloan T (2013). Muscle relaxant use during intraoperative neurophysiologic monitoring. J Clin Monit Comput.

[22] Gaddam S, Santhi V, Babu S, Chacko G, Baddukonda R, Rajshekhar V (2012). Gross and microscopic study of the filum terminale: does the filum contain functional neural elements?. J Neurosurg Pediatr.

[23] George T, Bulsara K, Cummings T (2003). The immunohistochemical profile of the tethered filum terminale. Pediatr NeuroSurg.

[24] Khealani B, Husain A (2009). Neurophysiologic intraoperative monitoring during surgery for tethered cord syndrome. J Clin Neurophysiology: Official Publication Am Electroencephalographic Soc.

[25] Quiñones-Hinojosa A, Gadkary C, Gulati M, von Koch C, Lyon R, Weinstein P, Yingling C (2004). Neurophysiological monitoring for safe surgical tethered cord syndrome release in adults. Surg Neurol.

[26] Varghese M, Olstorn H, Murrell W, Langmoen I (2010). Exploring atypical locations of mammalian neural stem cells: the human filum terminale. Arch Ital Biol.

[27] Arvidsson L, Fagerlund M, Jaff N, Ossoinak A, Jansson K, Hägerstrand A, Johansson C, Brundin L, Svensson M (2011). Distribution and characterization of progenitor cells within the human filum terminale. PLoS ONE.

[28] Bao N, Chen Z, Gu S, Chen Q, Jin H, Shi C (2007). Tight filum terminale syndrome in children: analysis based on positioning of the conus and absence or presence of lumbosacral lipoma. Child’s Nerv System: ChNS : Official J Int Soc Pediatr Neurosurg.

[29] Cornips E, Vereijken I, Beuls E, Weber J, Soudant D, van Rhijn L, Callewaert P, Vles J (2012). Clinical characteristics and surgical outcome in 25 cases of childhood tight filum syndrome. Eur J Pediatr Neurology: EJPN : Official J Eur Pediatr Neurol Soc.

