# Supplementary material for: The discrepancy in triggered electromyography responses between fatty filum and normal filum terminale
Source: BMC Surg. 2024 Feb 16;24:60. doi: 10.1186/s12893-024-02351-0 (PMC10874075; doi:10.1186/s12893-024-02351-0)

**Supplemental Content Legends**

Supplementary figure 1. Heatmap of triggered EMG in filum terminale with electromyographical responses (Before sorting).

Supplementary figure 2. Immunohistochemical examination of fatty filum terminale (GFAP, S100).


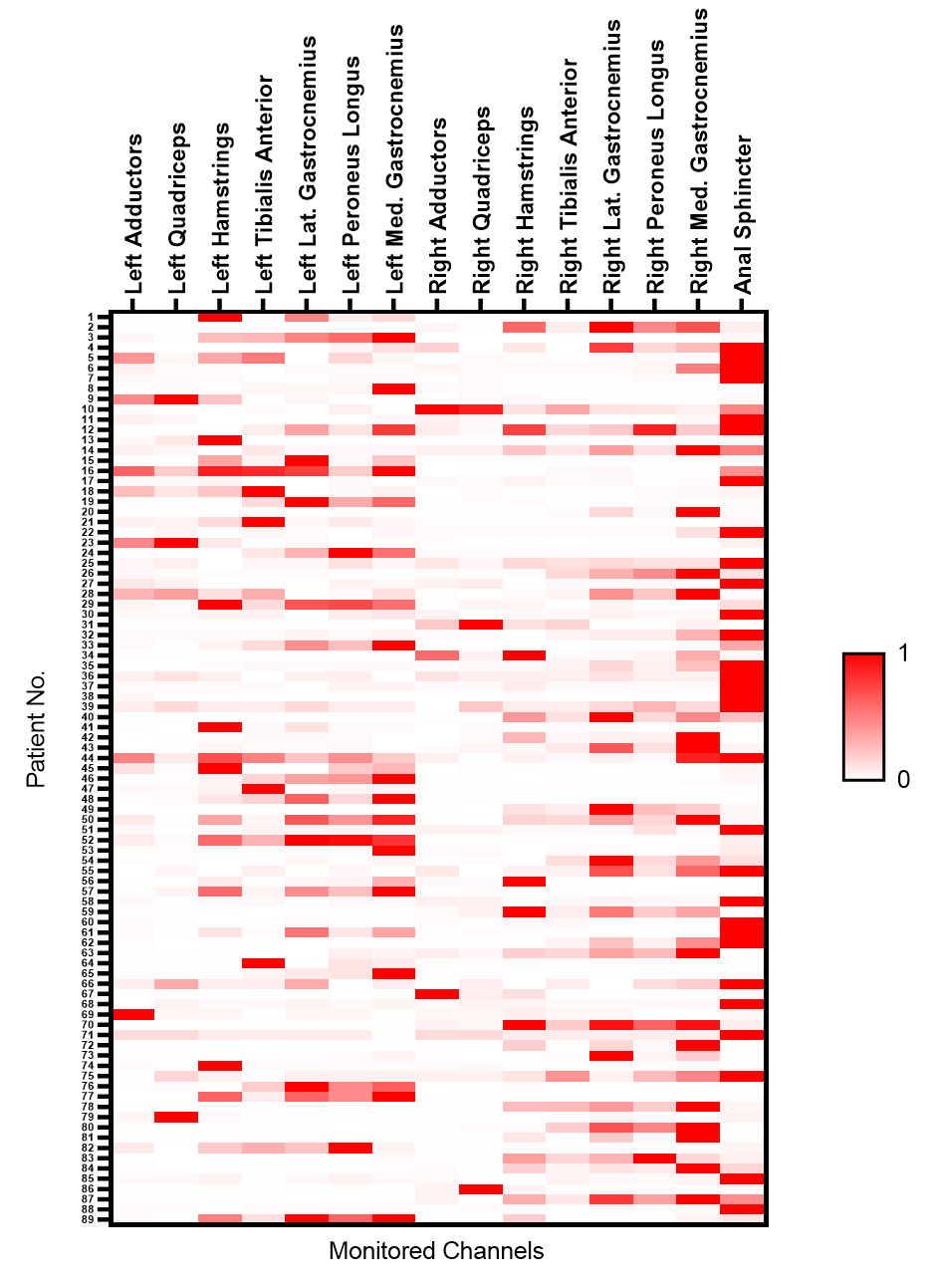


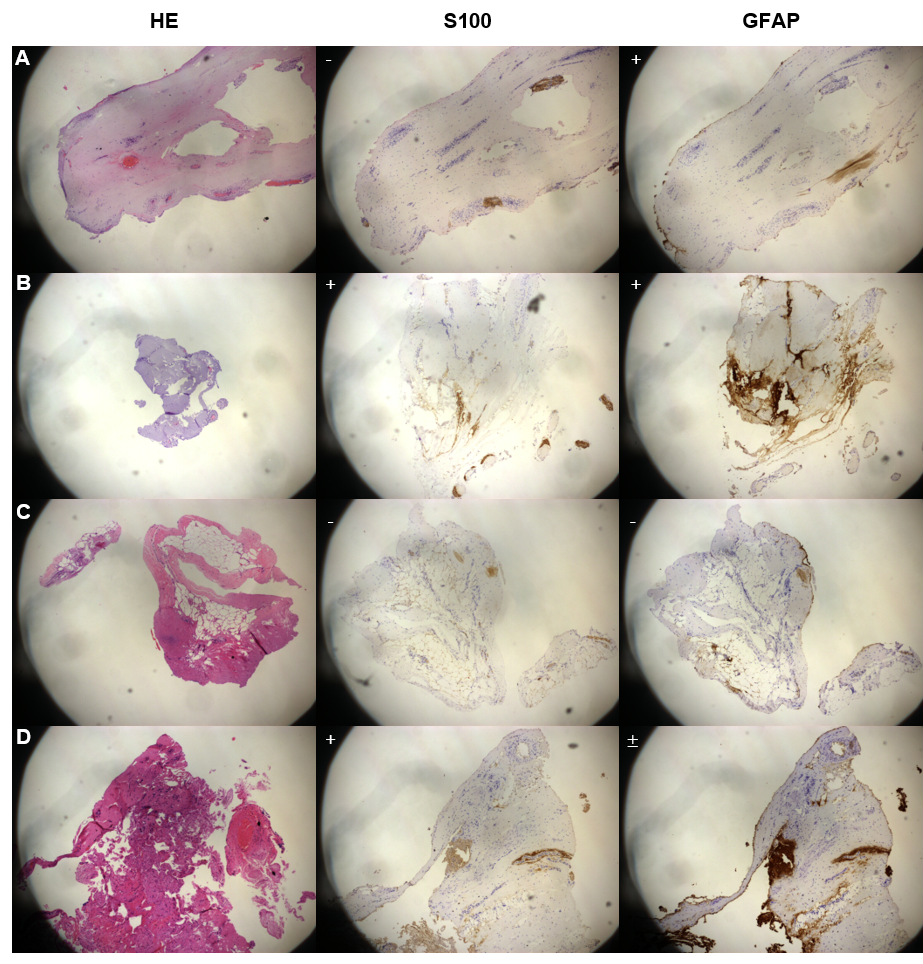

Supplement: Supplementary file 1 — Supplementary Material 1 [file 12893_2024_2351_MOESM1_ESM.docx]
